# Meta-population structure in a coral reef fish demonstrated by genetic data on patterns of migration, extinction and re-colonisation

**DOI:** 10.1186/1471-2148-8-248

**Published:** 2008-09-12

**Authors:** Line K Bay, M Julian Caley, Ross H Crozier

**Affiliations:** 1 School of Marine and Tropical Biology, James Cook University, Townsville, Qld 4811, Australia; 2 Australian Institute of Marine Science, PMB #3, Townsville MC, QLD 4810, Australia; 3 ARC Centre of Excellence for Coral Reef Studies, Townsville, Qld 4811, Australia and Australian Institute of Marine Science, PMB #3, Townsville MC, QLD 4810, Australia

## Abstract

**Background:**

Management strategies for coral reefs are dependant on information about the spatial population structure and connectivity of reef organisms. Genetic tools can reveal important information about population structure, however, this information is lacking for many reef species. We used a mitochondrial molecular marker to examine the population genetic structure and the potential for meta-population dynamics in a direct developing coral reef fish using 283 individuals from 15 reefs on the Great Barrier Reef, Australia. We employed a hierarchical sampling design to test genetic models of population structure at multiple geographical scales including among regions, among shelf position and reefs within regions. Predictions from island, isolation-by-distance and meta-population models, including the potential for asymmetric migration, local extinction and patterns of re-colonisation were examined.

**Results:**

*Acanthochromis polyacanthus *displayed strong genetic structure among regions (Φ_ST _= 0.81, P < 0.0001) that supported an equilibrium isolation-by-distance model (r = 0.77, P = 0.001). Significant structuring across the continental shelf was only evident in the northern region (Φ_ST _= 0.31, P < 0.001) and no evidence of isolation-by-distance was found within any region. Pairwise Φ_ST _values indicated overall strong but variable genetic structure (mean Φ_ST _among reefs within regions = 0.28, 0.38, 0.41), and asymmetric migration rates among reefs with low genetic structure. Genetic differentiation among younger reefs was greater than among older reefs supporting a meta-population propagule-pool colonisation model. Variation in genetic diversities, demographic expansion and population growth estimates indicated more frequent genetic bottlenecks/founder effects and subsequent population expansion in the central and southern regions compared to the northern one.

**Conclusion:**

Our findings provide genetic evidence for meta-population dynamics in a direct developing coral reef fish and we reject the equilibrium island and isolation-by distance models at local spatial scales. Instead, strong non-equilibrium genetic structure appears to be generated by genetic bottlenecks/founder effects associated with population reductions/extinctions and asymmetric migration/(re)-colonisation of such populations. These meta-population dynamics varied across the geographical range examined with edge populations exhibiting lower genetic diversities and higher rates of population expansion than more central populations. Therefore, coral reef species may experience local population reductions/extinctions that promote overall meta-population genetic differentiation.

## Background

Coral reefs are important ecosystems in ecological, evolutionary and socio-economic contexts but are under increasing threat from anthropogenic impacts [1,2]. The most effective conservation tool available to coral reef managers so far has been the use of individual or networks of Marine Protected Areas (MPAs) [2,3]. To maximise the effectiveness of MPAs information about the spatial population structure, patterns of connectivity and the stability of local populations within and among protected areas is required [3,4]. Genetic tools can provide valuable information about the scale, structure and stability of populations [5] when direct census estimates required to empirically demonstrating these processes are impractical to obtain [6,7]. The development and application of molecular markers to examine patterns of connectivity in coral reef organisms have increased greatly in recent years [5], but comparatively little attention has been placed on examining predictions from meta-population theory [8] despite the intuitive appeal of this approach in describing such systems. Here we present only the second examination of genetic meta-population dynamics in a coral reef fish in more than a decade and the first to use a highly variable and drift-sensitive molecular marker.

Genetic models of spatial structure have developed from Wright's original island model [9] into the stepping-stone, or isolation-by-distance models [10-13], and later into meta-population models [e.g. [14-19]]. The island model has played a central role in the development of population ecological and evolutionary theory because of its mathematical simplicity and tractability, but it makes many assumptions including equal population sizes, equal migration rates, discrete generation times, amongst others [20]. When these assumptions are met, populations should display similar genetic diversities, levels of sub-division and demographic histories [20]. The isolation-by-distance model relaxes these assumptions somewhat by allowing migration rates to be higher among populations in close proximity compared to more distant ones [20]. Both the island and isolation-by-distance models assume drift-migration equilibrium, which may not be met when migration rates are low and/or genetic bottlenecks are frequent [8].

In contrast to these island-based models, meta-population theory attempts to understand systems of evolutionarily ephemeral, genetically subdivided populations that persist through time via colonisation and migration from source populations [21-23]. Such populations are connected by migration rates that are high enough to rescue local populations from extinction, but low enough for genetic drift to generate measurable genetic differences among populations [22]. Meta-population dynamics can therefore be distinguished from island dynamics by low, variable levels of migration among populations. While earlier models assumed that migration was infrequent, and only re-colonised patches in which populations had gone extinct [14,15], it is becoming evident that migration rates may be asymmetrical [24] and can vary temporally [e.g. [25,26]], spatially [e.g. [27-29]] and behaviourally among individuals [e.g. [30-32]]. In turn, such variation in migration rates may generate a diversity of genetic signatures depending on the relative importance of each process [24]. A comparison of traditional (based on the island model) and coalescence-based analytical approaches that can separate overall genetic differentiation into reciprocal migration rates [33,34] should be able to illuminate the roles of migration and drift in establishing patterns of genetic differentiation among populations [8].

Theory suggests that the sources and rates of colonisation relative to subsequent migration are critical determinants of the evolution of the genetic structure of meta-populations [18,19,23]. In a meta-population with low levels of migration, the meta-population propagule-pool model predicts high genetic differentiation among populations if empty patches are colonised by individuals from a single source [18,19]. This high genetic differentiation results from genetic bottlenecks arising from founder effects of a few, genetically similar individuals. In contrast, under the meta-population migrant-pool model, low genetic differentiation among populations should result if extinct patches are colonised by many migrants from a larger number of source populations [18,19]. Because the colonisers are numerous and harbour greater genetic diversity, the re-colonised population will not experience a bottleneck, and because the genetic diversity is sampled from a range of sources, differentiation among populations will be decreased under this model. Under the propagule-pool model, populations will always display greater genetic differentiation than under an island model because of genetic bottlenecks associated with low and asymmetric colonisation rates. In contrast, greater genetic differentiation among populations will only occur under the migrant-pool meta-population compared to an island model if rates of colonisation and migration rates are low [18,19]. Consequently, meta-population dynamics can be distinguished from island dynamics by a strong but variable level of genetic structure among populations. Separating the effects of colonisation pattern and subsequent migration in meta-populations, however, is often difficult because the relative effects of colonisation and migration cannot be estimated from a single estimate of genetic differentiation [35]. If the propagule-pool model is operating, and if migration rates are low, then populations with younger coalescent histories should display greater genetic structure among each other compared to that found among older ones [35,23]. Therefore, it should be possible to distinguish different types of meta-population dynamics among genetically structured populations by patterns of genetic differentiation observed among populations that have experienced more recent population bottlenecks compared to those with older ones [36-40].

The effects of these meta-population dynamics on spatial genetic structure have typically been estimated in terms of genetic differentiation among populations, but may also be evident in patterns of genetic diversity and demographic history of local populations [23,40,41]. As such, important information about the role of local extinctions in a meta-population, and its importance in determining the geographic range of species, may be gained by examining patterns of genetic diversity and demographic history in sets of local populations [8,42,43]. In general, meta-population dynamics should reduce genetic diversity at the level of the meta-population and genetic diversity within the sub-populations compared to a panmictic population of equal size to the meta-population [40]. The relative magnitude of this difference, however, may vary greatly depending on the frequency and intensity of effective population size reductions among the sub-populations, and the mode of subsequent re-colonization and migration [[40] and references therein]. For example, reductions in genetic diversities may be large where reductions in the effective size of sub-populations are frequent and large, and if re-colonisation obeys a propagule-pool model. Coalescence times within sub-populations are also reduced under this scenario because of genetic bottlenecks associated with propagule-pool colonisation [40]. If sub-populations experience minor fluctuations in population size, or if colonisation obeys a migrant-pool model, where colonisers originate from a range of populations, sub-populations may not experience genetic bottlenecks and genetic diversities may not be affected to a measurable extent [23,40,41].

Fishes on coral reefs occupy a naturally fragmented environment where patches of suitable reef habitat are surrounded by unsuitable habitat such as open sand and deep water making them amenable to analysis under a meta-population framework. At present, however, we know little about the presence, spatial extent and genetic consequences of meta-populations dynamics in marine systems [[8], but see [44]]. Species with short, or non-existent larval durations generally display considerable genetic structure across small spatial scales [45-48] and have, therefore, the potential for genetic meta-population dynamics. Coral reef fishes generally display large effective population sizes [49] and many marine fishes are characterised by relatively shallow population genetic structures reflecting genetic bottlenecks associated with Pleistocene climate variation [reviewed by [50,51]]. Genetic bottlenecks following post-Pleistocene extinctions of local populations have previously been regarded as unimportant in the population dynamics of coral reef fishes [44,52]. Recent studies, however, have uncovered a diversity of coalescent signatures operating at a range of temporal scales [e.g. [53-58]] suggesting an important role of demographic bottlenecks and local extinctions in the evolutionary ecology of coral reef fishes.

*Acanthochromis polyacanthus *is a common fish on Australia's Great Barrier Reef (GBR) and lacks a dispersive larval phase. This life-history trait, coupled with the physical history of the GBR, and the sensitivity of mitochondrial molecular markers to drift, provides an opportunity to evaluate the potential importance of meta-population dynamics to the evolution of genetic structure on small spatial scales in a natural marine system. Previous investigations of *A. polyacanthus*, as well as the presence of several colour morphs on the GBR, suggest that sufficient time has elapsed since colonisation of the GBR began by this species for it to have evolved genetic differences among populations separated by small geographic distances [46,57,59,60]. Here we examine if and how the genetic structure of this species varies at two spatial scales (i.e. within and among regions). Using conventional genetic estimates of fixation, we test whether the genetic structure of this species is best described by equilibrium-island, or isolation-by-distance models. Next, we examine the evidence for meta-population dynamics in *A. polyacanthus *by evaluating spatial differences in migration rates and conformation to predictions from the propagule and migrant-pool re-colonisation models. Finally, we evaluate the role of extinction by comparing patterns of genetic diversity and demographic history among reefs and regions.

## Results

### Genetic diversities among regions and reefs

**Figure 1:**
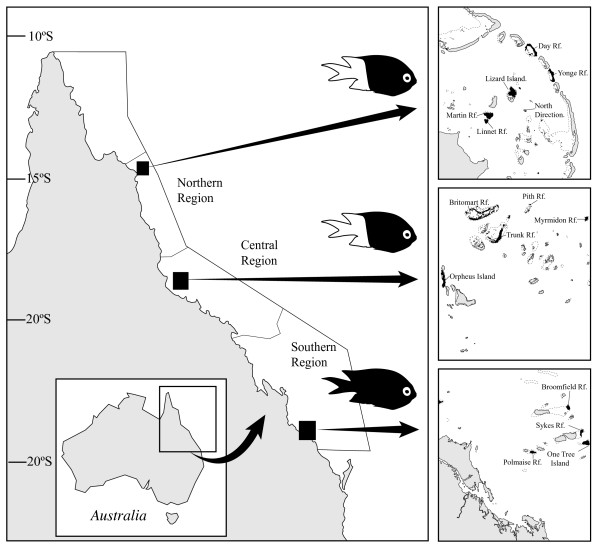
**Sampling locations of *Acanthochromis polyacanthus *on the Great Barrier Reef**. Fish illustrations indicate the distribution and sampling of colour morphs.

A region of 356 bases of the mtDNA control region I was obtained from a total of 283 individuals collected from 15 reefs in three regions (Figure 1). The average base frequencies were AT biased (A = 0.41, T = 0.40, C = 0.07, G = 0.12) as commonly observed in fish mtDNA [61,62]. The ts/tv ratio was 1.53:1 for all samples combined. Haplotype diversities were very high when summed over all populations (total ± SD = 0.97 ± 0.0003) and did not differ significantly among regions (Kruskal – Wallis = 0.187, df = 2, p = 0.91). Each region contained one or two reefs with significantly lower haplotype diversities than the rest (i.e., North = YON, Central = TRU, south = OTI and SYK, Table 1). Nucleotide diversities were high overall (total ± SD = 0.066 ± 0.37), and varied significantly among regions (Kruskal – Wallis Test = 10.64, df = 2, p = 0.005). Reefs in the northern region displayed the highest and most variable nucleotide diversities, whereas, nucleotide diversities were lower among locations within the central and southern locations (Table 1).

**Table 1:** Locations and genetic diversities of the 15 populations of *Acanthochromis polyacanthus *sampled by this study.

Region/Shelf	Location (abbreviation) Latitude; Longitude	N	Haplotype diversity (± SD)	Nucleotide diversity (± SD)
North		122	0.92 (0.013)	(0.035 ± (0.018)

Outer	Yonge Reef (YON) 14°37S; 145°37E	20	0.621 (0.063)	0.008 (0.005)
	Day Reef (DAY) 14°31S; 145°33E	22	0.788 (0.068)	0.015 (0.008)
Mid	Lizard Island (LIZ) 14°40S; 145°28E	20	0.826 (0.056)	0.033 (0.017)
	North Direction (NDR) 14°44S; 145°30E	19	0.778 (0.064)	0.04 (0.021)
Inner	Martin Reef (MAR) 14°45S; 145°20E	21	0.719 (0.1)	0.013 (0.007)
	Linnet Reef (LIN) 14°47S; 145°21E	20	0.816 (0.058)	0.041 (0.02)

Central		92	0.94 (0.009)	0.011 (0.006)

Outer	Pith Reef (PIT) 18°13S; 147°02E	21	0.81 (0.05)	0.007 (0.004)
	Myrmidon Reef (MYR) 18°16S; 147°23E	17	0.794 (0.078)	0.005 (0.003)
Mid	Britomart Reef (BRI) 18°14S; 146°39E	19	0.778 (0.072)	0.004 (0.003)
	Trunk Reef (TRU) 18°23S; 146°40E	14	0.604 (0.15)	0.002 (0.002)
Inner	Orpheus Island (ORP) 18°37S; 146°29E	21	0.752 (0.086)	0.003 (0.002)

South		69	0.83 (0.036)	0.007 (0.004)

Outer	One Tree Island (OTI) 23°30S; 152°05E	21	0.486 (0.124)	0.001 (0.001)
Outer	Sykes Reef (SYK) 23°26S; 152°02E	16	0.608 (0.09)	0.002 (0.002)
Mid	Polmaise Reef (POL) 23°34S; 151°41E	13	0.923 (0.069)	0.002 (0.002)
Outer	Broomefield Reef (BRO) 23°16S; 151°57E	19	0.836 (0.087)	0.0003 (0.002)

Geographical coordinates, shelf position and abbreviations used throughout this paper are indicated for each location. Number of individuals sequenced (N), their haplotype and nucleotide diversities and standard deviations (SD) are indicated.

### Spatial population genetic structure among regions and reefs

**Figure 2:**
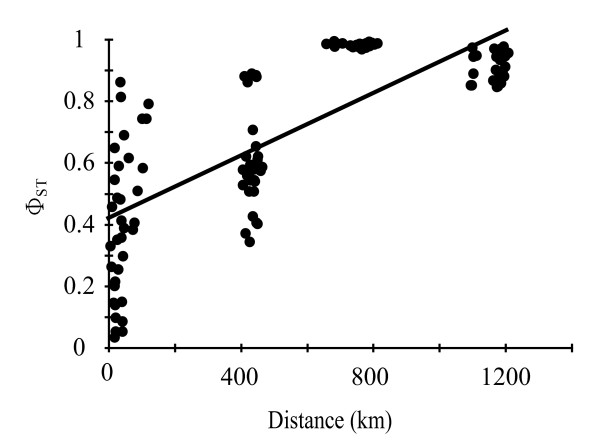
**Isolation-by-distance in *Acanthochromis polyacanthus *on the Great Barrier Reef**. Relationship between genetic differentiation and linear geographical distance. Φ_ST _= 0.00051 (0.00043 - 0.00058) km + 0.41 (0.36 - 0.47).

Strong and significant genetic structure was detected among regions by analysis of molecular variance (AMOVA) (Φ_ST _= 0.81, p < 0.0001, Table 2a). Significant structure was attributable to shelf position in the northern (Φ_ST _= 0.31, p < 0.001, Table 2b), but not in the central region (Table 2c). Results of the isolation-by-distance analyses were largely congruent with these results and significant correlation between geographical and genetic distances was only evident among regions (Φ_ST _vs. km: r = 0.77 p = 0.001; Figure 2). This correlation remained statistically significant when the number of reefs were reduced to six (two from each of the three regions) to make the number of comparisons similar to those used within regions (Φ_ST _vs. km: r = 0.91 p = 0.02; unpublished data). In contrast, genetic and geographic distances were not correlated within regions (P > 0.05 in all cases, unpublished data).

**Table 2:** Analysis of Molecular Variance within and among regions on the Great Barrier Reef

	d.f.	V	%	Fixation	p
	
a) Among regions					
Among regions	2	17.99	81.21	0.812	< 0.0001
Among populations within regions	12	1.93	8.71	0.463	< 0.0001
Within populations	282	2.23	10.0.8	0.90	< 0.0001
b) Within Northern Region					

Among shelves	2	2.102	29.74	0.297	0.015
Among populations within shelves	3	1.337	18.92	0.269	< 0.001
Within populations	116	3.628	51.34	0.487	< 0.001
c) Within Central Region					

Among shelves	2	0.125	9.88	0.099	0.201
Among populations within shelves	2	0.442	33.24	0.37	< 0.001
Within populations	87	0.722	56.88	0.431	< 0.001

a) Among regions (North, Central and South), b) within Northern region (among shelf position: Outer – DAY, YON; Mid: LIZ, NDR; Inner: LIN, MAR) c) within Central region (among shelf position: Outer – MYR, PIT; Mid: BRI, TRU; Inner: ORP). V = Variance component, % = percent variation explained, fixation = Φ_ST _and p = significance.

**Figure 3:**
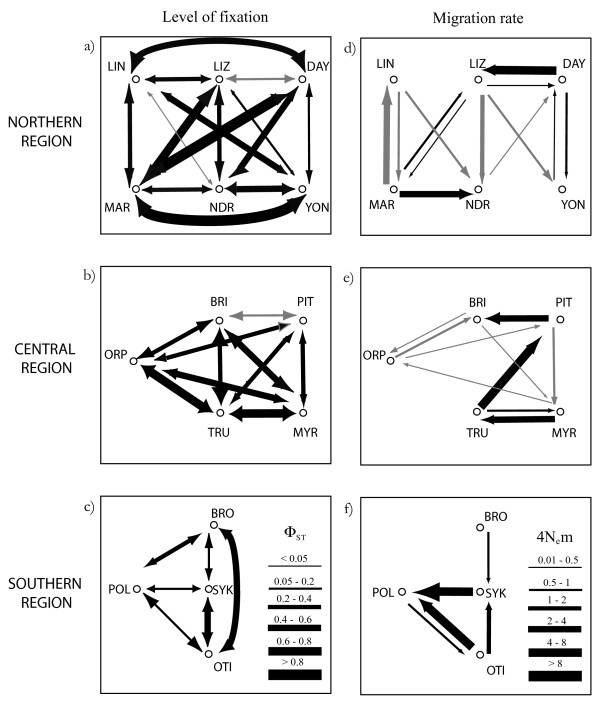
**Pairwise genetic distances and asymmetric migration rates among reefs within regions**. The thickness of arrows indicates the strength of genetic structure (Φ_ST_: a – c) or rate of migration (4N_e_m: d – f) and the colour indicates statistical difference. For pairwise genetic distances black arrows indicate estimates significantly different from 0. For migration rates black arrows indicate that reciprocal estimates were different (95% confidence intervals of did not overlap) and grey arrows indicate that reciprocal estimates were not different (95% confidence intervals of estimates overlapped).

Pairwise Φ_ST _estimates were significantly greater than 0 in more than 97% of all pairwise comparisons. Levels of genetic differentiation differed among reefs but the range of differentiation among reefs was similar within each of the three regions (Kruskal – Wallis Test = 1.21 df = 2, p = 0.55, Figure 3a – c, see Additional file 1). There was substantial variation in migration rates among reef samples and regions (Figure 3d – f). Migration rates were generally low (4N_e_m mostly < 1) and the frequency of significantly different reciprocal rates was higher in the southern region (i.e. 4N_e_m (x to y) vs. 4N_e_m (y to x): north = 23%, central = 20% and southern = 42%) (Figure 3d – f). All regions were characterised by one or two migration rates being significantly higher (4N_e_m ~ 4) than all other estimates. The level of fixation was greater between reef samples with more recent population expansions compared to the level of fixation among reef samples with older population expansions providing tentative support for the meta-population propagule-pool model of re-colonisation (Table 3).

**Table 3:** Level of genetic differentiation among older and younger populations.

Comparison	Older	Younger
Central region	PIT – BRI	ORP-TRU
	0.05	0.689
	PIT-MYR	
	0.357	
	MYR – BRI	
	0.401	
Southern region	POL-BRO	SYK-OTI
	0.297	0.459

Level of genetic differentiation is indicated by pairwise Φ_ST _values. Age of populations was estimated by the 95% CI of τ where younger populations were defined as those that did encompass 0 and older ones as those that did not encompass 0. Location abbreviations follow Table 1.

### Historical population demography among regions and reefs

Log-likelihood ratio tests indicated that the fit of the sudden expansion and constant population size models could be distinguished in 11 of the 18 comparisons (with near significance in one further reef sample) (Table 4). Support for sudden expansion was found in both the central and southern region including three central and one southern reef sample. In contrast, support for the constant population size model was found in one (or two if considering the near significant value) northern, one central and two southern reef samples (Table 4). The mean number of pairwise differences among regions corroborated these differences (Kruskal-Wallis Test = 8.04, df = 2, p = 0.018), and ranged from 0.55 in the southern, 1.95 in the central and 9.25 in the northern regions (Table 4). Likewise the age of population expansion (τ) was significantly different among regions (Kruskal-Wallis Test = 7.52, df = 2, p = 0.023) and ranged from 0.7 in the southern region, 5.2 in the central region and 3.1 in the northern region. The lower bound of the 95% confidence interval of τ could not be distinguished from 0 in the southern region although substantial uncertainty was associated with all estimates (95% CI: south = 0 – 3.4; central 2.4 – 7.8; north = 0.5 – 30.5). Estimates of population expansion (g) also varied significantly among regions (Kruskal-Wallis Test = 8.08, df = 2, p = 0.018) and were greater in the southern region (1936 ± SE = 71.3), intermediate in the central region (331 ± SE = 46.1), and close to 0 in the northern region (38 ± SE = 16.1). These differences among regions were largely supported by Fu's F_s _and R_2 _neutrality tests with the southern region displaying significant departures from neutrality in both indices indicating population expansion, the central region displaying some indication of population expansion when based on the Fs (although not significant after multiple test correction) but not using the R_2 _index and the northern region displaying non-significant values in both indices (Table 5).

**Table 4:** Demographic histories of *Acanthochromis polyacanthus *among reefs and regions using mismatch analysis.

Region or reef sample	Mismatch	SSD (obs/con)	SSD (obs/exp)	Log-likelihood ratio	p
North	9.25	0.060	0.085	4.78	0.09

DAY	4.14	0.081	0.085	0.71	0.87
YON	2.63	0.342	0.432	8.73	0.033
LIZ	8.96	0.079	0.078	0.12	0.98
NDR	9.11	0.142	0.187	3.25	0.36
LIN	10.88	0.136	0.515	17.30	0.0006
MAR	3.78	0.123	0.150	2.87	0.41

Central	1.95	0.095	0.005	27.44	0.008

MYR	1.10	0.040	0.256	0.93	0.019
PIT	2.22	0.192	0.139	19.65	0.0002
TRU	0.71	0.100	0.031	10.77	0.013
BRI	1.06	0.131	0.030	11.12	0.011
ORP	1.06	0.058	0.042	6.58	0.005

South	0.55	0.0027	0.0025	11.8	< 0.0001

POL	1.44	0.056	0.074	24.92	< 0.0001
BRO	0.74	0.008	0.011	15.72	0.0013
OTI	0.38	0.002	0.663	1.25	0.74
SYK	0.69	0.122	0.046	10.96	0.012

Mean number of pairwise differences (mismatch) and sums of squared deviation (SSD) from observed (obs) under constant (con) and exponential expansion (exp) models, log-likelihood ratio of population model fits and p value (FDR = 0.031). Location abbreviations follow Table 1.

**Table 5:** Demographic histories of *Acanthochromis polyacanthus *among reefs and regions using neutrality tests.

Region or reef sample	Fs	p	R_2_	p
North	4.33	0.91	0.18	1.00

DAY	2.23	0.84	0.16	0.86
YON	6.64	1	0.26	1.00
LIZ	5.32	0.97	0.18	0.89
NDR	5.41	0.99	0.21	0.98
LIN	8.82	0.99	0.25	1.00
MAR	2.89	0.89	0.17	0.90

Central	-6.12	0.015	0.07	0.068

MYR	1.28	0.85	0.14	0.39
PIT	0.11	0.57	0.16	0.71
TRU	-4.08	0.001	0.10	0.004
BRI	-1.02	0.028	0.13	0.40
ORP	1.43	0.82	0.13	0.38

South	-16.54	0.000	0.03	< 0.000

POL	-0.86	0.33	0.12	0.11
BRO	-3.34	0.03	0.10	0.08
OTI	-2.14	0.07	0.13	0.35
SYK	0.23	0.63	0.17	0.55

Fu's Fs and R_2 _neutrality statistic and p values (Fu's and R_2 _FDR = 0.008). Location abbreviations follow Table 1.

**Figure 4:**
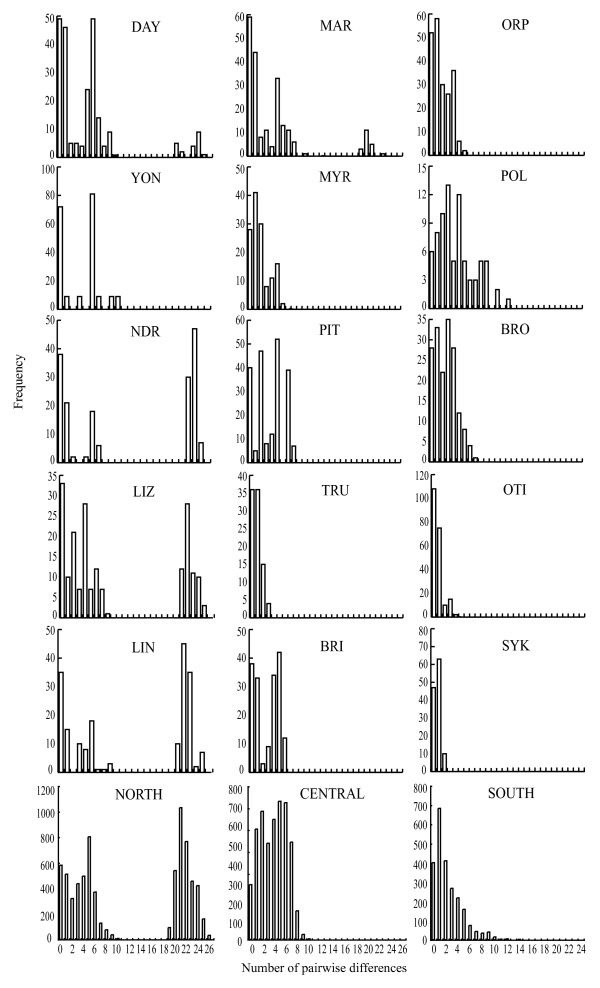
**Mismatch distributions of pairwise sequence differences in *Acanthochromis polyacanthus *among regions and reefs**. Panels represent the mismatch frequency distribution of individual reefs (n = 15) or regions (n = 3). Location abbreviations follow Table 1.

**Figure 5:**
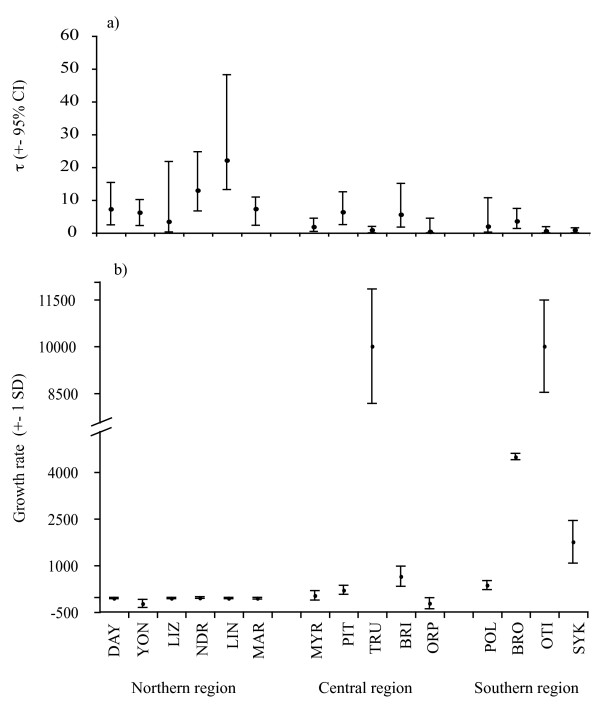
**Demographic expansion parameters of *Acanthochromis polyacanthus *among reefs on the Great Barrier Reef**. a) Expansion parameter (τ) from mismatch analysis and b) exponential growth rate (*g*). Location abbreviations follow Table 1.

Three types of mismatch distributions could be distinguished (Figure 4). Five reef samples in the northern region (DAY, NDR, LIZ, LIN, MAR) were characterised by bimodal mismatch distributions (Figure 4) indicating the presence of two genetically divergent lineages (see Additional data file 2). As a consequence these populations displayed greater mismatch means and support for the constant population model was found in LIN (Table 4). Three reef samples (TRU, OTI, SYK) were characterised by narrow left skewed uni-modal mismatch distributions and low mismatch means and support for population expansion was obtained for two of these reefs (Figure 4, Table 4). The remaining populations (n = 7) displayed broader uni-modal mismatch distributions with larger mismatch means (Figure 4). Support for the expansion model was found in three central reefs (PIT, BRI, ORP); the constant population model was supported in three reefs (MYR, POL, BRO and near significant in YON) (Table 4). The age of population expansion (τ) followed a similar pattern to that of the mismatch means and error estimates from most reefs in all three regions overlapped to a great extent (Figure 5a). Greater values with large variances were observed in two northern reefs (NDR and LIN), lower and less variable estimates were found in one central location (TRU) and in two southern locations (OTI and SYK) (Figure 5a). The age of population expansion (τ) could not be distinguished from 0 in four locations: TRU and ORP in the central region and OTI and SYK in the southern region and the neutrality indices indicated population expansion in one of these (TRU) (Table 4 and 5). Population expansion rates varied significantly among reef samples (Figure 5b). All northern locations displayed negative growth rates close to 0. Reefs in the central region showed both positive and negative growth rates that were all close to 0 except TRU that displayed a highly positive value. The high mean regional growth rate in the southern region was contributed to by the high growth rates of three of the four southern reefs. The growth rates of these three reefs were greater than all other reefs analysed except one (TRU in the central region) (Figure 5b).

## Discussion

### Genetic structure among regions

The mtDNA marker used here revealed strong genetic structure among northern, central and southern populations of *A. polyacanthus *on the GBR. This result is consistent with previous findings of strong allozyme structure between northern region bi-coloured and southern region black morphs of this species [59,60] and indicates the presence of further strong structuring among bi-coloured morphs between northern and central regions. This pattern of genetic structure is consistent with large-scale water circulation patterns on the GBR, which are characterised by an offshore outer shelf bifurcation of the South Equatorial Current into a northern flowing Hiri and a southern flowing East Australian Currents around 16°S latitude [63,64] positioned between the Northern and Central regions examined here. At smaller spatial scales this inflow generates complex continental shelf current patterns modulated by winds, tides and local reef matrix densities [65]. Such cross-shelf currents differ among regions and are predominantly weaker and flow in an onshore direction in the northern region, are stronger and flow in an offshore direction in the central and southern regions of the GBR [65]. The genetic structure of *A. polyacanthus *was consistent with these cross shelf current patterns. Continental shelf effects were detected in the northern but not in the central region and a higher abundance of individuals in a second divergent lineage was found on more inshore northern reefs (Figure 4). Our analyses also indicate that the spatial structure among regions is best described by an isolation-by-distance model of dispersal in which genetic exchange is more likely among neighbouring locations than more distant ones (Figure 2). While patterns of isolation-by-distance have been reported at large spatial scales in marine organisms [e.g. [66,67]], our study reports one of very few examples of such dynamics at smaller spatial scales in a coral reef fish [see also [44,68]] and suggests that a migration-drift equilibrium may be met at this spatial scale [8,69].

### Genetic structure within regions

Strong genetic structure was evident within all three regions and was attributable to continental shelf position in the northern region (Table 2b), however, no evidence of isolation-by-distance was found within any of the regions. These results are consistent with a departure from migration-drift equilibrium and a greater importance of genetic drift over migration in structuring this species at smaller spatial scales [8]. It is possible, however, that reduced statistical power resulting from fewer reefs being compared within regions than between prevented the detection of isolation-by-distance within regions where it existed. A reduction in the number of reefs compared among regions to the number of reefs compared within regions detected a significant correlation between genetic and geographical distance. Although larger sample sizes within regions will be required to resolve this issue fully, this result indicates that our tests were powerful enough to detect isolation-by-distance even when fewer comparisons were included. Genetic differentiation among reefs within regions was comparable (Figure 3a – c) and generally very high. For example, Lizard Island (LIZ) and North Direction Island (NDR) are separated by less than 10 km but have a Φ_ST _value of 0.26 and Martin Reef (MAR) and Linnet Reef (LIN) are separated by less than 6 km and have a Φ_ST _value of 0.33. Such differentiation is among the highest recorded for any coral reef fish at such small spatial scales [e.g. [58,70,71]] and is, despite obvious difficulties in comparing differentiation based on different molecular markers and taxa, comparable to values obtained for many direct-developing coral reef organisms at similar spatial scales [45,47,48,57,72-75]. This finding suggests that the spatial patterns described by this study may be broadly applicable to many direct developing coral reef species.

### Asymmetric migration and re-colonisation of reefs within regions

Despite the strong genetic structure of *A. polyacanthus *among reefs, our analyses indicated that migration rates were substantial and asymmetric in between 20 – 40% of comparisons. These patterns indicate a departure from the island model (which assumes equal migration rates among all populations) and add to a growing number of examples documenting asymmetric migration rates using genetic evidence [e.g. [32]]. Variation in migration rates may affect the dynamics of meta-populations and spatial structure of species in several ways [22,26]. If habitat patches differ in quality and local populations differ in size, migration from larger source populations into smaller sink populations may facilitate their long-term persistence by rescuing them from extinction [24,26,27]. If such dynamics are spatially and temporally persistent they should be identifiable by low genetic differentiation, and high and mostly uni-directional migration from sources to sinks. The strength of genetic differentiation was largely congruent with the Bayesian estimates of migration (Figure 3) and insignificant or small pairwise Φ_ST _values were often (e.g. DAY – LIZ; BRI – PIT; NDR – MAR; OTI – POL; SYK – POL) but not always (e.g. NDR – LIN) associated with asymmetric migration rates. The direction of migration however, did not identify any obvious sources or sinks in *A. polyacanthus *since most potential sinks (reefs that received a higher number of immigrants) also produced substantial numbers of emigrants as expected for potential source reefs (e.g. LIZ, TRU, POL, OTI: Figure 3d – f). The patterns uncovered here may indicate that migration at local scales is a stochastic process, although further sampling would be required to fully determine this. A second process by which asymmetric migration can affect the spatial structure of a meta-population is through the re-colonisation of extinct patches [18,19,35]. Asymmetric colonisation events from a single patch such as those observed among some reefs here (e.g. MYR to TRU; POL to OTI) could result in more genetic structure among more recently (re)-colonised patches through the effect of genetic drift associated with such founder effects, but then erase this structure over time as more migrants are received (propagule-pool model). The strength of fixation (Φ_ST_) was greater between younger, more recently expanded populations compared to that found between reefs that expanded longer ago providing support for the colonisation model (Table 3). This study therefore adds to only a handful of investigations that have explored, and largely supported, the predictions from such meta-population colonisation models [reviewed by [35]]. This conclusion, however, is based on coalescence estimates with considerable variation and a small number of comparisons, and should, therefore, be interpreted with caution. It does imply, however, that population size reductions/extinctions and genetic bottlenecks/founder effects associated with re-colonisation of such reefs have the potential to increase the level of genetic structure in this meta-population, at least over relatively short evolutionary time scales.

### Genetic diversities and historical population demography

Genetic diversities recorded here were generally high and comparable with those reported for other coral reef fishes using the same mitochondrial marker [e.g. [51,71] but see [76]] supporting the general observation of high genetic diversity in many coral reef fish species [50]. Intra-specific variation in genetic diversities in *A. polyacanthus *was substantial both among reefs and regions and greater than those previously reported among reef fish populations separated by more than 17000 km [68], or taxonomically distinct species from different environments [51]. Our analyses also indicated consistent and substantial variation in historical population growth patterns of *A. polyacanthus *among reefs and regions on the GBR (Table 1, 4, 5, Figure 4 and 5). The central and southern regions, and many (although not all) reefs within them, were characterised by population expansion indicated by mismatch analysis and neutrality indices. In particular, the southern region, located close to the species' southern border, was characterised by lower mitochondrial genetic diversity and population expansion rates 6 – 19 times greater than the central and northern regions. These lower genetic diversities and higher population expansion rates could be the result of either demographic or spatial range expansions. Spatial range expansion is particularly worth considering given the proximity of the sampled reefs in the southern region to the geographical range limit of *A. polyacanthus*. The genetic signatures of demographic and range expansion models can be very similar [77] particularly when migration rates among sub-populations are high [78,79]. When migration rates are low, spatially expanding populations may display multi-modal mismatch distributions, similar to those expected under constant population size models [78]. While it is possible that both the central and southern regions represent a spatial range expansion of this species in a north to south direction along the GBR, we consider this unlikely for two reasons. First, the distribution of colour morphs and lineages within colour morphs were confined to regions. If north-to-south range expansion were occurring in this species, we would have expected colonisation of both northern lineages in the central region and colonisation of black and white lineages in the southern region. This was not the case (see Additional file 2). Second, we did not sample any multi-modal mismatch distributions in the central and southern regions as expected in spatially expanding species with relatively low migration rates (Figure 4). The demographic expansion model therefore appears a more parsimonious explanation for our results, although further sampling in the southern region would be required before these hypotheses can be definitively distinguished. In all, this suggests that genetic bottlenecks and founder effects arising through colonisation of new or extinct sub-populations may affect the meta-population dynamics towards the edges of a species' geographic range to a greater extent than within more centrally located regions [42,43].

There were substantial differences in the mismatch distributions and population growth rates among reefs within regions (Figure 4, 5, Table 4). Most reefs had comparably high genetic diversities (Table 1), low population expansion rates, and times that were significantly different from 0 (Figure 5). This provides evidence for constant population size and was further supported by mismatch analysis for four of these reefs (YON, MYR, BRO, POL). Three reefs Trunk (TRU), One Tree Island (OTI) and Sykes Reef (SYK) were characterised by low genetic diversities (Table 1), uni-modal, left-skewed mismatch distributions (Figure 4), high population growth rates and expansion times that could not be distinguished from 0 (Figure 5) all consistent with population expansion. Mismatch analysis indicated population expansion in a further three reefs (BRI, ORP, PIT) from the central region. In concert, these results provide evidence for recent population bottlenecks and/or local extinctions on these reefs. Four reefs in the northern region (NDR, LIZ, LIN, MAR) had very high nucleotide diversities and bimodal mismatch distributions that were most likely the result of constant population sizes (as indicated by mismatch analysis for LIN, Table 4) and the presence of approximately equal numbers of individuals from two differentiated lineages at these locations (Figure 4, Additional file 2). The presence and maintenance of two or more divergent lineages across relatively small spatial scales is emerging as a feature of many coral reef organisms [51,80-82]. The dynamic history of coral reefs associated with sea-level fluctuations from the mid Miocene to the end of the Pleistocene has been implicated as the major evolutionary force promoting divergence and subsequent coalescence in species with high dispersal potential [e.g. [51,76,82]]. Because of the limited dispersal potential of *A. polyacanthus*, deep genetic divergences may evolve among locations in the absence of sea-level fluctuations and significant genetic structure has previously been found between GBR, Coral Sea and Melanesian populations of this species [[57,60], Additional file 2]. It is therefore likely that colonisation events from these locations may be the source of the second lineage prominent on the inshore northern reefs highlighting the potential for long distance dispersal in this brooding species. The absence of this second lineage from several northern, all central and southern reefs, however, indicates that this is likely to be a rare and/or recent event.

## Conclusion

The population genetic structure of *A. polyacanthus *on the GBR contained significant variation consistent with an equilibrium isolation-by-distance model at larger spatial scales and a non-equilibrium meta-population dynamics model at smaller ones. Meta-population dynamics were evident at smaller spatial scales indicated by the high levels of population structure (consistent with propagule-pool re-colonisation), asymmetric migration rates, variation in genetic diversities and historical demography parameters. The maintenance of strong genetic structure despite considerable migration rates and a signature of population expansion in many reef samples indicate that local population size fluctuations and extinctions may play an important role in generating genetic structure in this coral reef fish. While meta-population models provide an intuitive framework to describe the dynamics of fragmented eco-systems and were supported by genetic evidence in the direct developing species examined here, the general application of this model to other coral reef organisms remains unclear. The brooding habit of *A. polyacanthus *is unusual, however, examples of strong natal homing and significant genetic structure at local scales are increasingly being documented in more typical, coral reef fishes with larval dispersal [e.g. [5,83]]. Furthermore, the diversity of demographic histories displayed by coral reef fishes, including the results presented here, indicates substantial potential for non-equilibrium population genetic dynamics that may vary across the species' geographical range. Future population genetic studies of coral reef organisms should therefore incorporate local and regional sampling regimes of species with different life histories. Doing so will further enhance our understanding of the role of meta-population dynamics and local population fluctuations in the ecology and evolution of coral reef associated species.

## Methods

### Study species and sampling locations

*A. polyacanthus *were collected from 15 back-reefs from 3 regions on the Great Barrier Reef during 2003 and 2004 (Table 1, Figure 1). This species is very common and show not apparent variation in abundance on reefs either along or across the GBR [84,85]. The geographic distribution of this species extends from 15°N to 26°S http://www.fishbase.org and the southern region sampled here was therefore near the southern limit of this species. *A. polyacanthus *is polymorphic with a southern black morph, and a black and white morph in the central and northern regions of the reef. Fish were captured using small hand spears or baited fence nets and hand nets and were transported either alive or on ice to the nearest shore where genetic samples (fin clips) were taken and preserved in 80% EtOH. Genetic effects of continental shelf position, inner, middle and outer, were examined among replicate reefs in two regions (i.e. north and central). Because the southern region contains no true inner- and mid-shelf zones, the genetic structure in this region was explored using pairwise genetic distances

### DNA extraction and amplification

Genomic DNA was extracted from approx 0.25 cm^2 ^of fin tissue (re-hydrated by several TE washes) using a modified CTAB extraction procedure [[86], excluding the phenol-chloroform step] and re-suspended in 50 μl of TE. Concentrated DNA stock was diluted 1:50 yielding a final DNA concentration of approximately 50 ng/μL. A 400 base-pair region of the mitochondrial control region (hyper variable region I, HVR I) was amplified using the specific forward primer (dLoopF 5'-CATATATGTRTTATCAACATTA-3') and the universal primers CR-E H16498 (5'-CCTGAAGTAGGAACCAGATG-3') [71]. PCR reactions were carried out on a PE Applied Biosystems 9700 in 25 μl containing 1× PCR Buffer (Promega), 3.5 mM MgCl_2_, 200 μM each dNTP, 0.4 μM each primer, 10 ng template DNA and 0.1 unit of *Taq Polymerase *(Promega). Amplification using the polymerase chain reaction (PCR) was conducted with a cycling profile of 30 s at 94°C, 45 s at 48°C and 60 s at 72°C for 30 cycles. The cycling profile was flanked by an initial 2 min denaturing step (94°C) and a 10 min terminal extension phase (72°C). PCR products were cleaned up using PCR clean up columns (Qiagen) and re-suspended in 20 μL of ddH2O. Two μL of the purified product was sequenced in the forward and reverse direction using a dyenamic ET dye terminator kit (Megabase) chemistry (Amersham Biosciences). Sequence products were purified using Sephadex G-50 columns. Labelled extension products were sequenced on a Megabase 1000 (Amersham Biosciences). Representative sequences have been deposited in a public database [GenBank: DQ199666 – DQ199947].

### Data analysis

The control region sequences were aligned and edited using Sequencher 4.2 (GeneCodes Corp. Michigan USA) and ESEE [87]. The best model of nucleotide substitution was determined using Modeltest 3.5 [88] and PAUP* 4.0b10 [89]. The hierarchical likelihood tests and Akaike Information Criteria agreed that the Tamura and Nei model [90] with γ = 0.3012 fitted the data best (-LogLikelihood = 1220.65; AIC = 2453.30). This model and rate heterogeneity estimate was used in all following analyses of population genetic structure. Base frequencies and the ts/tv ratio from all sampled fish combined were calculated using Modeltest. The role of saturation was explored by comparing the topology of neighbour joining trees (implemented in PAUP*) including and excluding transitions. All individuals retained membership in the same major clades and transitions were included in all further analyses.

### Population Genetic Structure

Estimates of mitochondrial haplotype and nucleotide diversity [91-93] and their associated standard deviations were calculated using Arlequin 3.11 [94] for each reef and region. Statistical differences in genetic diversity among regions were determined using Kruskal-Wallis tests implemented in SPSS 16.

Hierarchical population genetic structure of *A. polyacanthus *among regions and reefs was explored using AMOVA with 1000 permutations [95,96] implemented in Arlequin. Pairwise genetic distances among reefs were calculated using Arlequin and a false discovery rate to correct for multiple tests (Benjamini-Hochberg) was applied to all pairwise comparisons [97].

Differences in levels of migration among reefs were investigated further using Migrate 1.7.6.1 [33,34]. This program calculates reciprocal migration rates (i.e., 4N_e_m from a to b, and vice versa) using a coalescence maximum likelihood approach (Markov Chain Monte Carlo with Hastings Metropolis importance sampling) and assumes constant mutation rates and equal effective population sizes. Because of the molecular divergences detected by phylogenetic and AMOVA analyses, Migrate was run on each geographical region separately. Reciprocal migration rates were interpreted as different when their 95% confidence intervals did not overlap. Extensive sampling regimes including 10 short chains sampled 10,000 times each and 5 long chains sampled 100,000 each were averaged over 5 replicates. Migrate was implemented on a SGI Origin 3800 computer in the James Cook University High Performance Computing Facility using a ts/tv ratio of 1.53 (estimated by Modeltest). Earlier versions of Migrate had problems with convergence of estimates, migration estimates and their associated profile likelihoods in low signal data [98]. Here we used a newer version of Migrate with high signal data and found no evidence of lack of convergence as repeated runs were highly consistent using the implemented sampling strategy. We also found congruence of migration estimates with conventional estimates of population structure.

Genetic distances were estimated using the conventional genetic distance estimator, Φ_ST_, and geographical distances among locations were calculated using Vincenty's inverse method http://www.ga.gov.au/nmd/geodesy/datums/distance.jsp. Correlations between genetic and geographical distances were tested using a Mantel test (1,000 permutations) of both log-transformed and non-transformed data following [100] and implemented in GenAlEx 6 [101]. A false discovery rate to correct for multiple tests (Benjamini-Hochberg) was applied to all pairwise comparisons [97]. Log transformation did not affect the overall results and therefore, only non-transformed kms versus Φ_ST _are presented here.

### Demographic History

Demographic histories were explored using mismatch analysis in Arlequin and DnaSP [100] using 1000 bootstrap replicates. These analyses computed the distribution of pairwise nucleotide differences to that expected under population models of constant and sudden expansion and assume that sub-populations are panmictic. The best fit of models was determined using log-likelihood ratio tests. The sums of square deviations (SSD) from the observed mismatch distributions were calculated for each of the models and log-likelihoods calculated following the methodology outlined in [102]. The statistical significance of log-likelihood ratios was adjusted using FDR as above and when different, the model with the lowest SSD was accepted. The age of population expansion was estimated by τ = 2 μt, where μ = the mutation rate and t = generation time. τ values were not converted to absolute years because of uncertainty associated with estimating mutation rates in fishes [103] and because comparisons were relative among regions and reefs. Differences in τ values were compared among regions using a Kruskal-Wallis test. Predictions from the meta-population re-colonisation models were examined by comparing estimates of genetic differentiation among older and younger reefs. Relative ages of reef samples were defined using the time of population expansion estimates (τ). Younger reefs were defined by having τ confidence intervals that could not be distinguished from the present (i.e. 95% CI of τ included 0). Older reefs were defined by having τ confidence intervals that did not encompass the present (i.e. 95% CI of τ did not include 0). Following this methodology, replicate younger and older reefs could only be compared in the central and southern regions because the 95% CI of τ did not include 0 in any northern reefs. Because of the uncertainty associated with τ estimates and the relatively low number of reefs compared here, these results should be interpreted with caution.

The exponential population growth parameter (*g*) was calculated among reefs and regions using a maximum likelihood coalescence approach implemented in Fluctuate 1.4 [104]. This approach assumes that subpopulations are panmictic, that population structure, growth, immigration and recombination rates have remained constant throughout the lifespan of the underlying coalescent tree [104]. A search strategy, each 10000 steps long using ten short chains, sampling every 20^th ^step, followed by ten long chains each of 20000 steps sampled every 20^th ^step, gave consistent results among runs and was used in all analyses. Estimates of *g *were compared among regions using a Kruskal-Wallis test. We calculated Fu's F_s _[105] and R_2 _[100] neutrality indices using DnaSP because they are the most sensitive to population growth [106]. Significance level was corrected for multiple testing using FDR as above.

## Authors' contributions

LKB conceived and designed the study, collected the samples, carried out the molecular work, analysed the data and drafted the manuscript. MJC and RHC assisted with the design of the study, analysis and interpretation of results and the preparation of the manuscript. All authors read and approved the final manuscript

## Supplementary Material

Additional file 1 Pairwise genetic distances among reef samples. Pairwise Φ_ST _values are indicated in the lower diagonal and their associated p values are indicated in the upper diagonal (FDR = 0.049). Click here for file

Additional file 2 Tree of unique haplotypes. The phylogenetic structure of *A. polyacanthus *was explored using Bayesian inference implemented in MrBayes 3.0B4 [107]. The analysis included 92 unique haplotypes found in the 283 individuals discussed above, 10 black morph individuals from Great Keppel Island (23°10S; 150°57E) and three black and white morph individuals from the Solomon Islands (9°24S; 160°32E). The analysis was performed using a Markov Chain Monte Carlo search with four chains for one million generations. Trees were sampled every 100 generations and the first 100,000 generations were discarded as burn-in. The tree was out-group rooted using two closely related species, *Amphiprion melanopus *and *A. akindynos*. Credibility values were obtained from a majority rule consensus tree of the last 2000 trees and values greater than 90% are indicated on the major nodes of the tree. Click here for file

## References

[B1] Wilkinson C Status of coral reefs of the world 2002 Townsville, Australian Institute of Marine Science

[B2] Hughes TP Baird AH Bellwood DR Card M Connolly SR Folke C Grosberg R Hoegh-Guldberg O Jackson JBC Kleypas J Lough JM Marchall P Nystrom M Palumbi SR Pandolfi JM Rosen B Roughgarden J Climate change, human impacts, and the resilience of coral reefs Science 2003 301 929 933 12920289 10.1126/science.1085046

[B3] Palumbi SR Population genetics, demographic connectivity, and the design of marine reserves Ecol Appl 2003 13 S146 S158

[B4] Thorrold SR Jones GP Hellberg ME Burton RS Swearer SE Neigel JE Morgan SG Warner RR Quantifying larval retention and connectivity in marine populations with artificial and natural markers B Mar Sci 2002 70 291 308

[B5] Jones GP Planes S Thorrold SR Coral reef fish larvae settle close to home Curr Biol 2005 15 1314 1318 16051176 10.1016/j.cub.2005.06.061

[B6] Almany GR Berumen ML Thorrold SR Planes Jones GP Local replenishment of coral reef fish populations in a marine reserve Science 2007 316 742 744 17478720 10.1126/science.1140597

[B7] Hellberg ME Footprints on water: the genetic wake of dispersal among reefs Coral Reefs 2007 26 463 473

[B8] Hellberg ME Kritzer JP, Sale PF Genetic approaches to understanding marine metapopulation dynamics Marine Metapopulations 2006 Amsterdam, Academic Press 413 455

[B9] Wright S Evolution in Mendelian populations Genetics 1931 16 97 159 17246615 10.1093/genetics/16.2.97 PMC1201091

[B10] Wright S Isolation by distance Genetics 1943 28 114 138 17247074 10.1093/genetics/28.2.114 PMC1209196

[B11] Kimura M Solution of a process of random genetic drift with a continous model Proc Natl Acad Sci U S A 1955 41 3 144 150 16589632 10.1073/pnas.41.3.144 PMC528040

[B12] Kimura M Weiss GH The stepping stone model of population structure and the decrease of genetic correlation with distance Genetics 1964 49 561 576 17248204 10.1093/genetics/49.4.561 PMC1210594

[B13] Weiss GH Kimura M A mathematical analysis of the stepping-stone model of genetic correlation J Appl Prob 1964 2 129 149

[B14] Levins R Gerstenhaber M Extinction Some mathematical problems in biology 1970 Providence, American Mathematical Society 75 107

[B15] Slatkin M Gene flow and genetic drift in a species subject to frequent local extinctions Theor Popul Biol 1977 12 253 262 601717 10.1016/0040-5809(77)90045-4

[B16] Slatkin M Gene Flow in Natural Populations Annu Rev Ecol Syst 1985 16 393 430

[B17] Slatkin M Gene flow and the geographic structure of natural populations Science 1987 236 787 792 3576198 10.1126/science.3576198

[B18] Wade MJ McCauley DE Extinction and recolonization – their effects on the genetic differentiation of local populations Evolution 1988 42 995 1005 10.1111/j.1558-5646.1988.tb02518.x 28581169

[B19] Whitlock MC McCauley DE Some population genetic consequences of colony formation and extinction – genetic correlations within founding groups Evolution 1990 44 1717 1724 10.1111/j.1558-5646.1990.tb05243.x 28567815

[B20] Hedrick PW Genetics of Populations 2003 Massachusetts, Jones and Bartlett Publishers

[B21] Hanski IA Metapopulation Ecology 1999 Oxford, Oxford University Press

[B22] Hanski IA Gilpin ME Meta-population biology. Ecology, genetics and evolution 1997 San Diego, Academic Press

[B23] Pannell JR Charlesworth B Effects of metapopulation processes on measures of genetic diversity Philos Trans R Soc Lond B Biol Sci 2000 355 1404 1851 1864 11205346 10.1098/rstb.2000.0740 PMC1692908

[B24] Stacey PB Taper M Environmental variation and the persistence of small populations Ecol Appl 1992 2 18 29 10.2307/1941886 27759195

[B25] Harrison S Gilpin ME, Hanski IA Local extinction in a metapopulation context: An empirical evaluation Metapopulation dynamics: Empirical and theoretical investigations 1991 London, Academic Press 73 88

[B26] Stacey PB Johnson VA Taper ML Hanski IA, Gilpin ME Migration within metapopulations: The impact upon local population dynamics Metapopulation biology. Ecology, genetics and evolution 1997 San Diego, Academic Press 267 291

[B27] Pulliam HR Sources, sinks and population regulation Am Nat 1988 132 652 661

[B28] Hanski IA Gyllenberg M Two general metapopulation models and the core-satellite species hypothesis Am Nat 1993 142 17 41

[B29] Valone TJ Brown JH Effects of competition, colonisation and extinction on rodent species diversity Science 1995 267 880 883 7846530 10.1126/science.7846530

[B30] Aars J Ims RA Population dynamics and genetic consequences of spatial density-dependent dispersal in patchy populations Am Nat 2000 155 252 265 10686164 10.1086/303317

[B31] Blundell GM Ben-David M Groves P Bowyer RT Geffen E Characteristics of sex-biased dispersal and gene flow in coastal river otters: implications for natural recolonisation of extirpated populations Mol Ecol 2002 11 289 303 11928704 10.1046/j.0962-1083.2001.01440.x

[B32] Fraser DJ Lippe C Bernatchez L Consequences of unequal population size, asymmetric gene flow and sex-biased dispersal on population structure in brook charr (*Salvelinus fontinalis*) Mol Ecol 2004 13 67 80 14653789 10.1046/j.1365-294x.2003.02038.x

[B33] Beerli P Felsenstein J Maximum-likelihood estimation of migration rates and effective population numbers in two populations using a coalescent approach Genetics 1999 152 763 773 10353916 10.1093/genetics/152.2.763 PMC1460627

[B34] Beerli P Felsenstein J Maximum likelihood estimation of a migration matrix and effective population sizes in n subpopulations by using a coalescent approach Proc Natl Acad Sci U S A 2001 98 4563 4568 11287657 10.1073/pnas.081068098 PMC31874

[B35] Giles BE Goudet J Hanski IA, Gilpin ME A case study of genetic structure in a plant metapopulation Metapopulation biology. Ecology, genetics and evolution 1997 San Diego, Academic Press 429 454

[B36] Olivieri I Couvet D Gouyon PH The genetics of transient populations – research at the metapopulation level Trends Ecol Evol 1990 5 207 210 21232356 10.1016/0169-5347(90)90132-W

[B37] Gilpin ME The genetic effective size of a metapopulation Biol J Linn Soc 1991 42 165 175

[B38] McCauley DE Genetic consequences of local population extinction and recolonization Trends Ecol Evol 1991 6 5 8 21232411 10.1016/0169-5347(91)90139-O

[B39] Harrison S Hastings A Genetic and evolutionary consequences of metapopulation structure Trends Ecol Evol 1996 11 180 183 21237801 10.1016/0169-5347(96)20008-4

[B40] Pannell JR Charlesworth B Neutral genetic diversity in a meta-population with recurrent local extinction and recolonisation Evolution 1999 53 664 676 10.1111/j.1558-5646.1999.tb05362.x 28565620

[B41] Pannell JR Coalescence in a meta-population with recurrent local extinction and recolonisation Evolution 2003 57 949 961 12836814 10.1111/j.0014-3820.2003.tb00307.x

[B42] Lennon JJ Turner JRG Connell D A meta-population model of species boundaries Oikos 1997 78 486 502

[B43] Holt RD Keitt TH Alternative causes for range limits: a meta-population perspective Ecol Lett 2000 3 41 47

[B44] Planes S Galzin R Bonhomme F A genetic metapopulation model for reef fishes in oceanic islands: the case of the surgeonfish, *Acanthurus triostegus* J Evol Biol 1996 9 103 117

[B45] Bernardi G Barriers to gene flow in *Embiotoca jacksoni*, a marine fish lacking a pelagic larval stage Evolution 2000 54 226 237 10937199 10.1554/0014-3820(2000)054[0226:BTGFIE]2.0.CO;2

[B46] Planes S Doherty PJ Bernardi G Strong genetic divergence among populations of a marine fish with limited dispersal, *Acanthochromis polyacanthus*, within the Great Barrier Reef and the Coral Sea Evolution 2001 55 2263 2273 11794786 10.1111/j.0014-3820.2001.tb00741.x

[B47] Bernardi G Vagelli A Population structure in Banggai cardinalfish, *Pterapogon kauderni*, a coral reef species lacking a pelagic larval phase Mar Biol 2004 145 803 810 10.1016/j.margen.2009.01.001 21798164

[B48] Hoffman EA Kolm N Berglund A Arguello JR Jones AG Genetic structure in the coral-reef-associated Banggai cardinalfish, *Pterapogon kauderni* Mol Ecol 2005 14 1367 1375 15813777 10.1111/j.1365-294X.2005.02538.x

[B49] Palumbi SR Genetic divergence, reproductive isolation and marine speciation Annu Rev Ecol Syst 1994 25 547 572

[B50] Grant WS Bowen BW Shallow population histories in deep evolutionary lineages of marine fishes: insights from sardines and anchovies and lessons for conservation Heredity 1998 89 415 426

[B51] Fauvelot C Bernardi G Planes S Reductions in the mitochondrial DNA diversity of coral reef fish provide evidence of population bottlenecks resulting from Holocene sea-level change Evolution 2003 57 1571 1583 12940362 10.1111/j.0014-3820.2003.tb00365.x

[B52] Kritzer JP Sale PF Kritzer JP, Sale PF The metapopulation ecology of coral reef fishes Marine metapopulations 2006 Amsterdam, Academic Press 31 68

[B53] Thacker C Thompson AR Roje DM Shaw EY New expansions in old clades: population genetics and phylogeny of *Gnatholepis *species (Teleostei:Gobioidei) in the Pacific Mar Biol 2008 153 375 385

[B54] Craig MT Eble JA Robertson DR Bowen BW High genetic connectivity across the Indian and Pacific Oceans in the reef fish *Myripristis berndti *(Holocentridae) Mar Ecol-Prog Ser 2007 334 245 254

[B55] Bowen BW Muss A Rocha LA Grant WS Shallow mtDNA coalescence in Atlantic pygmy angelfishes (genus Centropyge) indicates a recent invasion from the Indian Ocean Heredity 2006 97 1 12 16394255 10.1093/jhered/esj006

[B56] Bowen BW Bass AL Muss A Carlin J Robertson DR Phylogeography of two Atlantic squirrelfishes (Family Holocentridae): exploring links between pelagic larval duration and population connectivity Mar Biol 2006 149 899 913

[B57] van Herwerden L Doherty PJ Contrasting genetic structures across two hybrid zones of a tropical reef fish, *Acanthochromis polyacanthus *(Bleeker 1855) J Evol Biol 2006 19 239 252 16405595 10.1111/j.1420-9101.2005.00969.x

[B58] Dudgeon CL Gust N Blair D No apparent genetic basis to demographic differences in scarid fishes across the continental shelf of the Great Barrier Reef Mar Biol 2000 137 1059 1066

[B59] Doherty PJ Mather P Planes S *Acanthochromis polyacanthus*, a fish lacking larval dispersal, has genetically differentiated populations at local and regional scales on the Great Barrier Reef Mar Biol 1994 121 11 21

[B60] Planes S Doherty PJ Genetic and color interactions at a contact zone of *Acanthochromis polyacanthus*: a marine fish lacking pelagic larvae Evolution 1997 51 1232 1243 10.1111/j.1558-5646.1997.tb03970.x 28565481

[B61] Wolstenholme DR Animal mitochondrial DNA: structure and evolution Int Rev Cytol 1992 141 173 216 1452431 10.1016/s0074-7696(08)62066-5

[B62] McMillan WO Palumbi SR Rapid rate of control region evolution in Pacific butterflyfishes (Chaetodontidae) J Mol Evol 1997 45 473 484 9342395 10.1007/pl00006252

[B63] James MK Armsworth PR Mason LB Bode L The structure of reef fish metapopulations: modelling larval dispersal and retention patterns Proc Biol Sci 2002 269 1505 2079 2086 12396481 10.1098/rspb.2002.2128 PMC1691134

[B64] Bode M Bode L Armsworth PR Larval dispersal reveals regional sources and sinks in the Great Barrier Reef Mar Ecol-Prog Ser 2006 308 17 25

[B65] Brinkman R Wolanski E Deleersnijder E McAllister F Skirving W Oceanic inflow from the Coral Sea into the Great Barrier Reef Estuar Coast Shelf S 2001 54 655 668

[B66] Palumbi SR Grabowsky G Duda TF Geyer L Tachino N Speciation and population genetic structure in tropical Pacific sea urchins Evolution 1997 51 1506 1517 10.1111/j.1558-5646.1997.tb01474.x 28568622

[B67] Planes S Fauvelot C Isolation by distance and vicariance drive genetic structure of a coral reef fish in the Pacific Ocean Evolution 2002 56 378 399 11926506 10.1111/j.0014-3820.2002.tb01348.x

[B68] Bay LK Choat JH van Herwerden L Robertson DR High genetic diversities and complex genetic structure in an Indo-Pacific tropical reef fish (*Chlorurus sordidus*): evidence of an unstable evolutionary past? Mar Biol 2004 144 757 767

[B69] Hellberg ME Stepping-stone gene flow in the solitary coral *Balanophyllia elegans*: equilibrium and nonequilibrium at different spatial scales Mar Biol 1995 123 573 581

[B70] Doherty PJ Planes S Mather P Gene flow and larval duration in seven species of fish from the Great Barrier Reef Ecology 1995 76 2373 2391

[B71] Bay LK Crozier RH Caley MJ The relationship between gene flow and pelagic larval duration in eight pomacentrid fish species on the Great Barrier Reef Mar Biol 2006 149 1247 1256

[B72] Hellberg ME Dependence of gene flow on geographical distance in two solitary corals with different larval dispersal capabilities Evolution 1996 50 1167 1175 10.1111/j.1558-5646.1996.tb02357.x 28565289

[B73] Ayre DJ Hughes TP Genotypic diversity and gene flow in brooding and spawning corals along the Great Barrier Reef, Australia Evolution 2000 54 1590 1605 11108587 10.1111/j.0014-3820.2000.tb00704.x

[B74] Ayre DJ Hughes TP Climate change, genotypic diversity and gene flow in reef-building corals Ecol Lett 2004 7 273 278

[B75] Underwood JN Smith LD van Oppen MJH Gilmour JP Multiple scales of genetic connectivity in a brooding coral on isolated reefs following catastrophic bleaching Mol Ecol 2007 16 771 784 17284210 10.1111/j.1365-294X.2006.03187.x

[B76] Klanten OS Choat JH van Herwerden L Extreme genetic diversity and temporal rather than spatial partitioning in a widely distributed coral reef fish Mar Biol 2007 150 659 670

[B77] Ibrahim KM Nichols RA Hewitt GM Spatial patterns of genetic variation generated by different forms of dispersal during range expansion Heredity 1996 77 282 291

[B78] Ray N Currat M Excoffier L Intra-Deme Molecular Diversity in Spatially Expanding Populations Mol Biol Evol 2003 20 1 76 86 12519909 10.1093/molbev/msg009

[B79] Excoffier L Patterns of DNA sequence diversity and genetic structure after a range expansion: lessons from the infinite-island model Mol Ecol 2004 13 853 864 15012760 10.1046/j.1365-294x.2003.02004.x

[B80] Knowlton N Sibling species in the sea Annu Rev Ecol Syst 1993 24 189 216

[B81] Barber PH Palumbi SR Erdmann MV Moosa MK Biogeography: a marine Wallace's line? Nature 2000 406 692 693 10963585 10.1038/35021135

[B82] Bernardi G Holbrook SJ Schmitt RJ Crane NL Genetic evidence for two distinct clades in a French Polynesian population of the coral reef three-spot damselfish *Dascyllus trimaculatus* Mar Biol 2003 143 485 490

[B83] Gerlach G Atema J Kingsford MJ Black K Miller-Sims V Smelling home can prevent dispersal of reef fish larvae Proc Natl Acad Sci U S A 2007 104 3 858 863 17213323 10.1073/pnas.0606777104 PMC1783404

[B84] Williams DM Patterns in the distribution of fish communities across the central Great Barrier Reef Coral Reefs 1982 1 35 43

[B85] Williams DM Baker JT, Carter RM, Sammarco PW, Stark KP Longitudinal and latitudinal variation in the structure of reef fish communities The inaugural Great Barrier Reef conference 1983 Townsville, James Cook University Press 265 270

[B86] Sambrook J Russell DW Molecular Cloning: A Laboratory Manual 2001 Europe, CSHL Press

[B87] Cabot E Beckenbach AT Simultaneous editing of multiple nucleic acid and protein sequences with ESEE Comput Appl Biosci 1989 5 233 243 2766009 10.1093/bioinformatics/5.3.233

[B88] Posada D Crandall KA Modeltest: testing the model of DNA substitution Bioinformatics 1998 14 817 818 9918953 10.1093/bioinformatics/14.9.817

[B89] Swofford DL Phylogenetic Analysis Using Parsimony (*and other methods) 1998 Sunderland, Sinauers Associates

[B90] Tamura K Nei M Estimation of the number of nucleotide substitutions in the control region of mitochondrial DNA in humans and chimpanzees Mol Biol Evol 1993 10 512 526 8336541 10.1093/oxfordjournals.molbev.a040023

[B91] Tajima F Evolutionary relationship of DNA sequences in finite populations Genetics 1983 105 437 460 6628982 10.1093/genetics/105.2.437 PMC1202167

[B92] Tajima F Takahata N, Clark AG Measurement of DNA polymorphism Mechanisms of Molecular Evolution. Introduction to molecular paleopopulation biology 1993 Tokyo, Japan Scientific Press

[B93] Nei M Molecular Evolutionary Genetics 1987 New York, Colombia University Press

[B94] Excoffier L Laval LG Schneider S Arlequin ver. 3.0: An integrated software package for population genetics data analysis Evol Bioinformatics Online 2005 1 47 50 PMC2658868 19325852

[B95] Weir BS Cockerham CC Estimating *F*-statistics for the analysis of population structure Evolution 1984 38 1358 1370 10.1111/j.1558-5646.1984.tb05657.x 28563791

[B96] Excoffier L Smouse PE Quattro JE Analysis of molecular variance inferred from metric distances among DNA haplotypes: application to human mitochondrial DNA restriction data Genetics 1992 131 479 491 1644282 10.1093/genetics/131.2.479 PMC1205020

[B97] Benjamini Y Hochberg Y Controlling the false discovery rate: A practical and powerful approach to multiple testing J R Stat Soc B 1995 57 289 300

[B98] Abdo Z Crandall KA Joyce P Evaluating the performance of likelihood methods for detecting population structure and migration Mol Ecol 2004 13 837 851 15012759 10.1111/j.1365-294x.2004.02132.x

[B99] Smouse PE Long JC Sokal RR Multiple Regression and Correlation Extensions of the Mantel Test of Matrix Correspondence Syst Zool 1986 35 627 632

[B100] Rozas J Sanchez-Delbarrio JC Messeguer X Rozas R DnaSP, DNA polymorphism analyses by the coalescent and other methods Bioinformatics 2003 19 2496 2497 14668244 10.1093/bioinformatics/btg359

[B101] Peakall R Smouse PE GenAlEx 6: genetic analysis in Excel. Population genetic software for teaching and research Mol Ecol Notes 2006 6 288 295 10.1093/bioinformatics/bts460 PMC3463245 22820204

[B102] Burnham KP Anderson DR Model Selection and Multimodel Inference: A Practical Information-Theoretic Approach 2002 New York, Springer

[B103] Ruzzante DE Walde SJ Gosse JC Cussac VE Habit E Zemlak TS Adams EDM Climate control on ancestral population dynamics: insight from Patagonian fish phylogeography Mol Ecol 2008 17 2234 2244 18363661 10.1111/j.1365-294X.2008.03738.x

[B104] Kuhner MK Yamato J Felsenstein J Maximum likelihood estimation of population growth rates based on the coalescent Genetics 1998 149 429 434 9584114 10.1093/genetics/149.1.429 PMC1460154

[B105] Fu YX Statistical tests of neutrality of mutations against population growth, hitchhiking and background selection Genetics 1997 147 915 925 9335623 10.1093/genetics/147.2.915 PMC1208208

[B106] Ramos-Onsins S Rozas J Statistical properties of new neutrality tests against population growth Mol Biol Evol 2002 19 2092 2100 12446801 10.1093/oxfordjournals.molbev.a004034

[B107] Huelsenbeck JP Ronquist F MR. BAYES: Bayesian inference of phylogenetic trees Bioinformatics 2001 17 754 755 11524383 10.1093/bioinformatics/17.8.754

