# Supplementary figures and images for: Meta-population structure in a coral reef fish demonstrated by genetic data on patterns of migration, extinction and re-colonisation
Source: BMC Evol Biol. 2008 Sep 12;8:248. doi: 10.1186/1471-2148-8-248 (PMC2553088; doi:10.1186/1471-2148-8-248)

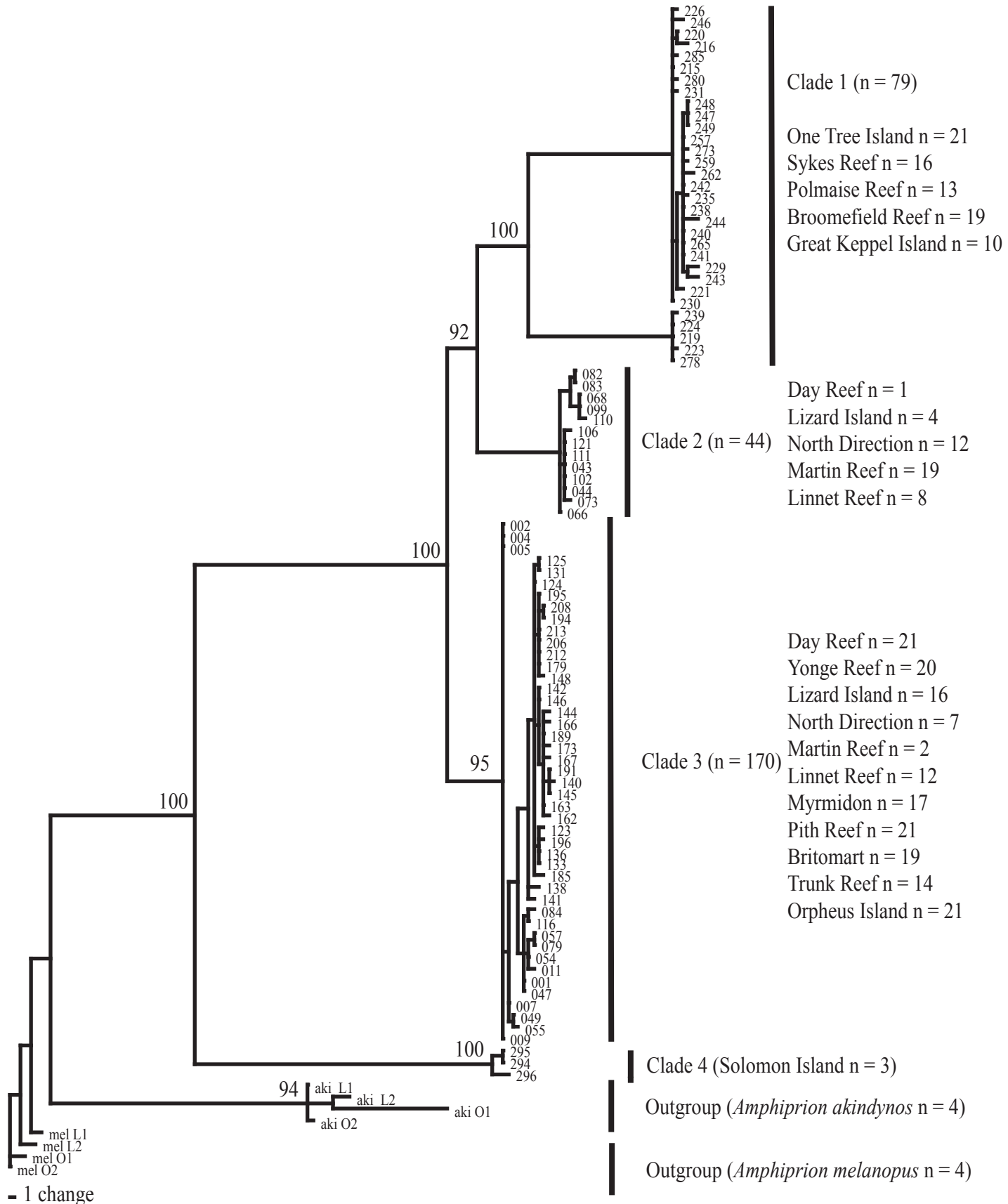

Supplement: Additional file 2 — Tree of unique haplotypes. The phylogenetic structure of A. polyacanthus was explored using Bayesian inference implemented in MrBayes 3.0B4 [107]. The analysis included 92 unique haplotypes found in the 283 individuals discussed above, 10 black morph individuals from Great Keppel Island (23°10S; 150°57E) and three black and white morph individuals from the Solomon Islands (9°24S; 160°32E). The analysis was performed using a Markov Chain Monte Carlo search with four chains for one million generations. Trees were sampled every 100 generations and the first 100,000 generations were discarded as burn-in. The tree was out-group rooted using two closely related species, Amphiprion melanopus and A. akindynos. Credibility values were obtained from a majority rule consensus tree of the last 2000 trees and values greater than 90% are indicated on the major nodes of the tree. [file 1471-2148-8-248-S2.pdf]
